# Commentary on: Muscle dysmorphia: Could it be classified as an addiction to body image?

**DOI:** 10.1556/JBA.3.2014.021

**Published:** 2014-12-18

**Authors:** JON E. GRANT

**Affiliations:** Department of Psychiatry & Behavioral Neuroscience, University of Chicago, Pritzker School of MedicineChicago, ILUSA

**Keywords:** addiction, compulsion, dysmorphia

## Abstract

This commentary addresses a recent article on the characterization of muscle dysmorphia as an addiction. The commentary examines the larger issue of the possible relationship of compulsions to addictions. It also questions whether understanding the heterogeneity within disorders may be a useful tactic to develop more targeted treatment approaches.

The article, “Muscledysmoprhia: Could it be classified as an addiction to body image”, by Foster, Shorter and Griffiths (2014) presents a compelling argument for reconceptualizing this type of body dysmorphic disorder as an addiction. Beyond the details of this particular disorder, however, the article alludes to a larger, more provocative look at the possible similarities between obsessional problems and addictions. Historically, the argument has been that obsessive compulsive problems are ego-dystonic and the behaviors reduce unwanted anxiety feelings. Conversely, addictive processes are thought to be at least initially ego-syntonic with behaviors in furtherance of reward. While this may be true for many individuals within each group, neuroscience and clinical research have now shown us that both obsessive and substance disorders are heterogeneous, that the elimination of a negative feeling is enormously rewarding for many individuals, that behaviors change over the course of illness, and therefore these categories of disorders are more fluid than initially thought (Cavedini, Gorini & Bellodi, 2006; Chamberlain et al., 2007; Figee et al., 2011; Jung et al., 2013).

Ultimately, an assessment of muscle dysmorphia’s relationship to established addictive disorders needs to consider the respective etiologies. Unfortunately, knowledge of many psychiatric disorders’ etiologies, particularly muscle dysmorphia, is not yet advanced enough to answer this question. Although not a direct measure of pathophysiology, responses to treatment may suggest different or shared neurobiologies, but here, too, the data are too limited to be helpful. Treatment for muscle dysmorphia has largely focused on pharmacological (selective serotonin reuptake inhibitors [SSRIs]) and cognitive behavioral therapy (CBT), but data regarding the efficacies of SSRIs and CBT for muscle dysmorphia are uncontrolled case series and reports (Pope, Phillips & Olivardia, 2000).

Before reclassifying muscle dysmorphia as an addiction, however, we might want to explore the idea that obsessions about body image might reflect a heterogeneous pathophysiology. Some individuals with muscle dysmorphia might be more similar to those with addictions, while others might be more similar to those with obsessive compulsive disorder or body dysmorphic disorder. The notion of muscle dysmoprhia as an addiction, although heuristically appealing, remains speculative and requires additional studies to examine its validity and appropriateness. In time, we might find out that the model proposed by Foster and colleagues is not simply limited to muscle dysmorphia but also applies to some individuals with obsessive compulsive disorder, anorexia nervosa, or other compulsive disorders.

The report by Foster and colleagues illustrates not only the similarities between muscle dysmorphia and addictive disorders but also the complexity of body image in general. Currently, there is no behavioral or pharmacological treatment for muscle dysmorphia supported by rigorous clinical trials. The distress of patients with muscle dysmorphia, the limited research performed to date, and the lack of available treatments with empirical validation highlight the importance of a reassessment of the disorder. Further exploration into the neurobiology of muscle dysmorphia is necessary to substantiate the hypotheses presented in this report. More comprehensive information, such as that which could be gleaned from neurobiological studies, has significant potential in advancing prevention and treatment strategies for not only muscle dysmorphia, but also other disorders characterized by body image obsessions or possibly obsessions in general.

